# Priority Areas for Adolescent Health Measurement

**DOI:** 10.1016/j.jadohealth.2020.12.127

**Published:** 2021-05

**Authors:** Regina Guthold, Ann-Beth Moller, Emmanuel Adebayo, Liliana Carvajal, Carolin Ekman, Lucy Fagan, Jane Ferguson, Howard S. Friedman, Mariame Guèye Ba, Ann Hagell, Kid Kohl, Peter S. Azzopardi

**Affiliations:** a Maternal, Newborn, Child and Adolescent Health and Ageing Department, WHO, Geneva, Switzerland; b UNDP/UNFPA/UNICEF/WHO/World Bank Special Programme of Research, Development and Research Training in Human Reproduction (HRP), Department of Sexual and Reproductive Health and Research, World Health Organization, Geneva, Switzerland; c Adolescent Health Unit, Institute of Child Health, University of Ibadan, Ibadan, Nigeria; d Division of Data Analytics Planning and Monitoring, Data and Analytics Section, UNICEF, New York, New York; e UN Major Group for Children and Youth, London, United Kingdom; f Technical Division, United Nations Population Fund, New York, New York; g University Cheikh Anta Diop of Dakar, Faculty of Medicine, Pharmacy and Odontology/Gynecology and Obstetrics Clinic, University Teaching Hospital A. Le Dantec, Dakar, Senegal; h Association for Young People's Health, London, United Kingdom; i Global Adolescent Health Group, Burnet Institute, Melbourne, Australia; j South Australian Health and Medical Research Institute, Aboriginal Health Equity theme, Adelaide, Australia; k Centre for Adolescent Health, Department of Paediatrics, University of Melbourne, Melbourne, Australia; l Independent Consultant, Sexual and Heproductive Health and Rights, Geneva, Switzerland; m Independent Consultant, Adolescent Health, Geneva, Switzerland

**Keywords:** Adolescence, Adolescent health, Burden of disease, Delphi, Global health, Measurement, Priority setting

## Abstract

**Purpose:**

We establish priority areas for adolescent health measurement and identify current gaps, aiming to focus resources on the most relevant data to improve adolescent health.

**Methods:**

We collected four critical inputs to inform priority setting: perspectives of youth representatives, country priorities, disease burden, and existing measurement efforts. Health areas identified from the inputs were grouped, mapped, and summarized according to their frequency in the inputs. Using a Delphi-like approach, international experts then selected core, expanded, and context-specific priority areas for adolescent health measurement from all health areas identified.

**Results:**

Across the four inputs, we identified 99 measurement areas relevant to adolescent health and grouped them under six domains: policies, programs, laws; systems performance and interventions; health determinants; health behaviors and risks; subjective well-being; and health outcomes and conditions. Areas most frequently occurring were mental health and weight status in youth representatives' opinions; sexual and reproductive health and HIV/AIDS in country policies and perspectives; road injury, self-harm, skin diseases, and mental disorders in the disease burden analysis; and adolescent fertility in measurement initiatives. Considering all four inputs, experts selected 33 core, 19 expanded, and 6 context-specific adolescent health measurement areas.

**Conclusion:**

The adolescent health measurement landscape is vast, covering a large variety of topics. The foci of the measurement initiatives we reviewed do not reflect the most important health areas according to youth representatives' or country-level perspectives, or the adolescent disease burden. Based on these inputs, we propose a set of priority areas to focus national and global adolescent health measurement.

**Implications and Contribution**

Using a systematic approach, this study identified priority areas for adolescent health measurement, as well as gaps. This supports countries and measurement initiatives to focus efforts and limited resources on the health areas of most potential utility for subsequent action to improve the health of adolescents.

See Related Editorial on p.836

Adolescents are key to sustainable development [1], and several global initiatives call for greater investment in their health [[1], [2], [3]]. Consequently, to track return of these investments, there has been a rapid proliferation of measurement efforts. However, these efforts have been poorly coordinated, resulting in duplication in some areas and persistent measurement gaps in others, along with the inconsistent definition, use and reporting of indicators, limiting comparability, and use of the data [[4], [5], [6]]. For example, a recent review revealed that more than 800 indicators—including variations of supposedly the same indicator—were used in adolescent reproductive health, but there were still data deficiencies for unmarried youth, adolescent boys, and very young adolescents and in specific areas such as abortion, nonheterosexual behavior, or fertility intentions [7].

There are several important aspects specific to this life stage to consider. First, adolescence is a developmental phase with rapid transitions in health needs that vary by sex, age, and sociodemographic factors [8]. Second, although the burden caused by mortality continues to play an important role from childhood throughout adolescence, the nonfatal disease burden and the future disease burden caused by risk factors require attention as adolescents develop [9]. Third, rapid epidemiological shifts are affecting adolescent health over time. More adolescents are now living in countries extending from a mainly infectious disease profile to an increasing burden of injury, violence, and noncommunicable diseases and—because of increasing inequities—in countries with the worst health situations, requiring targeted investments and measurement [10].

To address these issues and improve alignment and capacity for adolescent health measurement in countries, the World Health Organization (WHO) in collaboration with UN partners established the Global Action for Measurement of Adolescent Health (GAMA) Advisory Group (Panel 1) [11,12]. Given the breadth and complexity of the adolescent health measurement landscape, GAMA defined its first task to identify priority areas for adolescent health measurement globally [11,12], aiming to focus resources on the most important health issues and to identify measurement gaps.

Panel 1 **The Global Action for Measurement of Adolescent health (GAMA) Advisory Group**

**Establishment**

The GAMA Advisory Group was established in 2018 by WHO, with support from UNAIDS, UNESCO, UNFPA, UNICEF, UN Women, the World Bank Group, and the World Food Programme.

**Goals and objectives**

GAMA's overarching goal is to improve adolescent health measurement globally. Specific objectives include (1) the identification of priority areas for adolescent health measurement (the focus of this article); (2) the definition of a core set of adolescent health indicators to converge data collection and reporting efforts; (3) the development, promotion, and implementation of harmonized guidance for the measurement of these indicators to support countries and technical organizations in collecting useful data to track progress in the improvement of adolescent health. GAMA's work builds on existing measurement efforts and is undertaken in collaboration with countries and stakeholders to be relevant and recognized.

**Members**

The GAMA Advisory Group consists of 17 international adolescent health experts, including four young experts, from 14 countries covering all world regions. Members were selected through a competitive process, following an open call. Two WHO staff scored all applicants independently, based on set criteria considering their technical expertise and ensuring gender balance, geographic diversity, and coverage of the main health issues for adolescents across the group.

The objective of this article was to describe the approach used by GAMA to identify core areas for health measurement of all adolescents aged 10–19 years globally, as well as expanded and context-specific areas that may only be relevant in specific regions, income groups, or adolescent population subgroups.

## Methods

### Approach to priority setting

**Figure 1:**
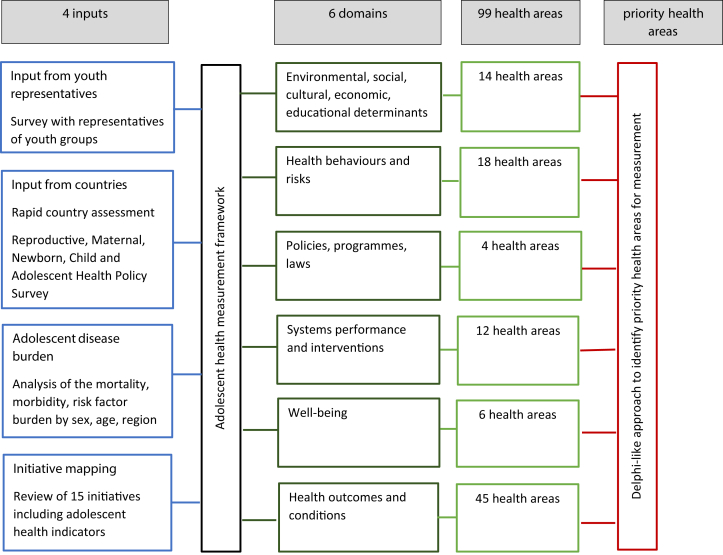
Approach to priority setting.

Our approach to priority setting was based on the principle of combining values (such as perspectives from stakeholders) and technical considerations (such as the disease burden or existing measurement efforts) [13] and was similar to earlier work on Indigenous adolescents [14]. First, we collected four inputs to reflect (1) perspectives of youth representatives; (2) priorities in countries; (3) the adolescent mortality, morbidity, and risk factor burden; and (4) inclusion of specific adolescent health areas in existing measurement initiatives. Second, we mapped all health areas collected through these inputs and aligned them with health areas of existing frameworks. To enable this, we used a broad structure of six domains forming our adolescent health measurement framework [2,15]. Third, we summarized the frequency of inclusion of each health area in the four inputs. Based on this summary and their expertise in the measurement of adolescent health, the 17 international experts of the GAMA Advisory Group [11,12] selected priority areas, using a Delphi-like approach [16] to reach consensus (Figure 1). The details of our approach are described below.

### Four inputs to inform priority setting

1. Input from youth representatives

To collect perspectives of people representing youth on what was most important for the health of adolescents, the four young experts of the GAMA Advisory Group (aged below 30 years) developed an online survey for 18- to 29-year-old representatives of youth-led organizations (referred to as “youth representatives”). Respondents were asked to choose up to five of 20 predefined health outcomes and up to 10 of 35 determinants influencing the health of adolescents. They could also propose other health topics not listed. Survey questions were reviewed by GAMA experts and UN representatives, pilot tested on a geographically diverse sample, and modified where feedback indicated that questions were not well understood. The final survey was available in the six UN languages (Arabic, Chinese, English, French, Russian, and Spanish). It was administered during July to September 2019 using the LimeSurvey online tool [17] and distributed through the Partnership for Maternal, Newborn, and Child Health, the UN Major Group for Children and Youth, UNICEF's Adolescent Development and Participation Regional Advisors, representatives of other UN organizations, and other youth networks. Further details are provided in the Appendix (p. 1–6). In addition, information from two recent reports deemed relevant were considered: “Health & Technology–What young people really think” [18] and “Our future, our health. Multi-country consultation with Young People on Primary Health Care” [19].

2. Input from countries

Input from countries was twofold: First, we analyzed data from 148 countries responding to the WHO Reproductive, Maternal, Newborn, Child and Adolescent Health (RMNCAH) Policy Survey [20], conducted between August 2018 and April 2019. This survey tracks country progress in adopting WHO recommendations in national health policies, strategies, and guidelines related to RMNCAH and is undertaken every 2–3 years. National responses are coordinated by the Ministry of Health focal points. Specifically, we analyzed the question: “Are adolescents cited as a specific target group for defined interventions/activities in a national policy/guideline for the following health issues?” by adding the number of positive responses for each of 14 health issues listed.

Second, a rapid assessment tool was developed by GAMA experts based on the Global AA-HA! [2] and distributed to adolescent health country professionals at two WHO regional adolescent health workshops (one in the WHO South-East Asia region and one in the WHO African region), and through networks of the GAMA experts. Respondents were asked to choose up to 10 health areas they thought most important for adolescent health measurement in their country from a predefined list and to propose additional health areas not listed. Further information on the RMNCAH Policy Survey analysis and the rapid assessment tool is provided in the Appendix (p. 7–13).

3. The adolescent burden of disease

Using the WHO Global Health Estimates [21], we analyzed (1) adolescent causes of death and (2) of nonfatal disease burden, as measured in Years Lost due to Disability. We also analyzed (3) adolescent risk factors for Disability-Adjusted Life Years (DALYs) and to account for future health loss and (4) adult risk factors for DALYs, using the Global Burden of Disease [22]. To account for variations in burden across different groups, these analyses were disaggregated by sex, age (for adolescents aged 10–14 and 15–19 years and for adults aged <50 years), and modified WHO region, whereby high-income countries were extracted from their region and combined in a separate group as in previous reports [2]. This resulted in 28 sex/age/regional groups for adolescents, and 14 for adults. For each sex/age/regional group, causes that contributed by more than 5% to the mortality or nonfatal disease burden of adolescents were listed. The risk factors that contributed by more than 5% to total adolescent or adult DALYs and that are prevalent and modifiable through interventions during adolescence were also included.

4. Existing measurement initiatives including adolescent health

To understand which areas of adolescent health are currently captured by existing measurement efforts and to identify gaps, we undertook a review of existing measurement initiatives and indicator compilations, identified through expert consultations and previous reports [5,10]. Experts consulted included GAMA experts, UN representatives, focal points from nine topic-specific WHO departments relevant for adolescent health, and WHO regional office focal points. To be included, an initiative or an indicator compilation needed to (1) include recommendations about adolescent health measurement; (2) propose at least one indicator specifically including “adolescent,” “youth,” or “young people” or include the entire or part of the adolescent age range 10–19 years; and (3) be global or regional in scope. All initiatives and indicator compilations identified were reviewed as to which specific adolescent health measurement areas they covered.

### Adolescent health measurement framework with key domains

We grouped all health areas collected through the four inputs under a broad structure of six domains forming our adolescent health measurement framework (Figure 1) [2,15]. Under each domain, we mapped the health areas across the four inputs. We then aligned them with health areas of existing frameworks for monitoring of health determinants [[23], [24], [25]]; the Institute for Health Metrics and Evaluation's Global Burden of Disease for health behaviors and risks [22]; the Global Strategy for Women's, Children's and Adolescents' Health 2016–2030 [26]; the Global Accelerated Action for the Health of Adolescents (AA-HA!) [2]; and the UNICEF Programmatic Guidance for the Second Decade: Programming with and for Adolescents [27] for the domain policies, programs, and laws; existing monitoring frameworks and measurement reviews for systems performance and interventions [28,29]; a systematic review of measurement scales for well-being [30]; and the WHO Global Health Estimates framework [21] for health outcomes and conditions.

### Delphi-like approach to select priority areas for adolescent health measurement

For the selection of priority areas for adolescent health measurement from all health areas identified across the six domains, the WHO Secretariat developed an online LimeSurvey [17]. To facilitate the selection, for each health area, the frequency of inclusion in the four inputs was summarized (Table 1), and individual results of the four inputs were also provided. Based on the inputs and their expertise in adolescent health measurement, independently of each other's responses and anonymously, the GAMA experts (Panel 1) were asked to review each area listed and select if they considered it to be a:

• “core measurement area” (relevant to all adolescents globally);
• “context-specific measurement area” (relevant only to adolescents of specific regions or specific subgroups); or
• “measurement area of currently limited importance” (an area that GAMA will not include in its current work).

**Table 1:** Adolescent health measurement areas with summarized inputs from youth representatives, countries, adolescent disease burden and measurement initiatives, and selected core, expanded, context-specific areas.

Adolescent health measurement area	Youth rep. Input	Country input	Disease burden	Measurement INITIATIVES	Selected area
**Social, cultural, economic, educational, environmental determinants of health**					
**Population (total and % adolescents)**		ME		ME	**Core**
**Education level/schooling status**	ME	HI		HI	**Core**
Employment status	ME	ME		HI	
Ethnicity		LO			Expanded
**Income level and poverty**	HI	ME		ME	**Core**
Being part of a vulnerable group (orphaned, out-of-school, migrant, minority etc)	ME	ME		ME	Expanded
Environment/pollution	HI	LO			Expanded
Disaster risk reduction				LO	
WASH (safe water source/sanitation, access to handwashing facility)	ME			LO	
Child marriage	LO	ME		HI	Context-specific
Child labor				LO	Context-specific
**Gender**	LO	ME		ME	**Core**
Social support	HI	ME		ME	Expanded
Social and cultural norms	LO				Context-specific
**Health behaviours and risks**					
High fasting plasma glucose			LO		
High systolic blood pressure			ME		
High LDL cholesterol			LO^a^		
**Weight status**	HI	ME	ME	HI	**Core**
**Alcohol use**	HI	HI	HI	HI	**Core**
**Substance use (other than alcohol and tobacco)**	HI	HI	HI	HI	**Core**
**Tobacco use**	HI	HI	ME	HI	**Core**
Gaming	LO				
Social media/internet	HI	ME		ME	Expanded
**Dietary behaviour**	HI	ME		ME	**Core**
**Physical activity**	HI	HI		HI	**Core**
Sedentary behaviour	HI			ME	Expanded
Sleep	HI				Expanded
**Bullying**		LO		ME	**Core**
**Sexual health**	HI	HI	ME^a^	HI	**Core**
**Reproductive health**	HI	HI		HI	**Core**
**Contraception**	HI	HI		HI	**Core**
Menstruation		LO		LO	
**Policies, programmes, laws**					
**Adolescent health policies/plans (availability, implementation, funding, M&E)**		LO		HI	**Core**
**Adolescent health protective laws (availability, implementation, funding, M&E)**				ME	**Core**
Adolescent health programmes (availability, implementation, funding, M&E)		LO			Expanded
Adolescents' participation in programming and planning		LO			Expanded
**Systems performance and interventions**					
**Health service availability and access**	HI	LO		ME	**Core**
**Health service quality**	HI	HI		LO	**Core**
Health service utilization and barriers	HI	ME		ME	Expanded
Health check-ups		LO			
**Immunization**	HI	LO		ME	**Core**
School health	ME	LO		ME	Expanded
Community health					
Health education	HI	ME		HI	Expanded
Training/education in adolescent health for professionals		LO		ME	
Social protection	ME			LO	Context-specific
Financial protection/health expenditure	HI	ME		LO	
**System for monitoring and surveillance of adolescent health**		LO		ME	**Core**
**Subjective well-being**					
Autonomy				LO	Expanded
Social connectedness	LO			LO	Expanded
Affect/feeling/emotion	LO			LO	
Life satisfaction				LO	
Meaning/achievement				LO	
Spirituality				LO	
**Health outcomes and conditions**					
**Mortality**					
**All-cause mortality**		ME		HI	**Core**
**Cause-specific mortality**		HI			**Core**
**Communicable, maternal, perinatal and nutritional conditions**					
**HIV/AIDS**	HI	HI	ME^a^	HI	**Core**
**STIs excluding HIV/AIDS**	HI	ME			**Core**
Tuberculosis	ME	ME	LO		
Lower respiratory infections	ME	ME	ME		
Diarrhoeal diseases	ME	ME	ME		
Meningitis	ME	ME	ME^a^		
Malaria	LO		LO^a^	LO	
Worms	LO			LO	
Maternal conditions			ME	ME	Expanded
Perinatal conditions			LO^a^		
Iron-deficiency	LO	ME	HI	ME	Context-specific
Vitamin A deficiency		LO	LO^a^		Context-specific
**Noncommunicable diseases**					
Leukaemia	**HI**	HI	HI	LO	
Brain and nervous system cancers	**HI**	HI	LO^a^	LO	
Cardiovascular diseases	HI	HI		LO	
Diabetes	HI	HI		LO	Expanded
Cirrhosis of the liver			LO^a^		
Sickle cell disorders and trait			LO^a^		
Congenital anomalies	LO		ME		
**Self-harm**	HI	HI	HI	ME	**Core**
**Anxiety disorders**	HI	HI	HI	LO	**Core**
**Depressive disorders**	HI	HI	HI	ME	**Core**
Childhood behavioural disorders	HI	HI	HI	LO	
Autism and Asperger syndrome			LO		
Stress/pressure	LO	LO			
Eye diseases and disorders	HI		LO^a^	LO	
Ear diseases and disorders	HI			LO	
Oral conditions	LO	LO		LO	
Asthma	LO	LO	ME		Expanded
Allergies	ME				
Skin diseases	HI		HI		
Migraine	LO		HI		
Back or neck pain	ME				
**Disability**	ME	LO			**Core**
Multi-morbidity		LO			
**Injuries (Unintentional and intentional)**					
**Road injury**	ME	HI	HI	HI	**Core**
Drowning	ME	LO	HI	HI	Expanded
Collective violence and legal intervention	HI	HI	ME^a^		Expanded
**Interpersonal violence**	HI	HI	HI	HI	**Core**
**Sexual violence**	HI	HI		HI	**Core**
**Gender-based violence**		HI		HI	**Core**
**Other health-related outcomes and conditions**					
**Adolescent fertility**		ME		HI	**Core**
Female genital mutilation/cutting	LO	LO		ME	

“HI”, “ME”, and “LO” represent high, medium, and low frequency, as follows: (1) youth representatives input: the number responses for each area (HI ≥200; ME 100-199; LO <100); (2) country input: score based on the number of mentions across the RMNCAH Policy Survey and the rapid assessment tool (HI score >5; ME score 3-5; LO score <3); (3) burden: the number of inclusions in any sex/age/regional group for causes of mortality, YLDs, or risk factors (HI included in >8 groups; ME included in 4-8 groups; LO included in <4 groups); (4) measurement initiatives: the number of inclusions of an area in existing measurement initiatives (HI >3 inclusions; ME: 2-3 inclusions; LO: 1 inclusion).

a Only included in one region.

After completion of Round 1, the survey results were synthesized by calculating the percentage of GAMA experts selecting “core,” “context-specific,” or “of limited importance” for each measurement area. Consensus on the category a measurement area fell into was based on agreement among the experts of at least 70% [31,32]. Measurement areas with agreement of at least 50% but less than 70% were further discussed. This discussion took place over email, and final consensus was reached during a face-to-face meeting of the GAMA experts.

## Results

### Four inputs to inform priority setting

1. Priority health areas identified by youth representatives

The 946 youth representatives (205 males, 556 females, 185 sex unknown) from 62 countries across all income groups and WHO regions responding to the online survey identified the most important health issues for adolescents as mental disorders/problems (n = 604), weight status (n = 448), interpersonal violence (n = 393), and skin diseases (n = 333). Most important determinants influencing the health of adolescents were support from parents (n = 413), social media/Internet (n = 393), knowledge about sexual health (n = 352), sleep (n = 330), alcohol use (n = 328), and tobacco use (n = 323; Table 2 and Appendix, p. 5–6).

2. Priority health areas in countries

**Table 2:** Top 10 health areas according to the different inputs

Rank	Youth representatives survey	RMNCAH policy survey	Rapid country assessment	Disease burden analysis	Inclusion in measurement initiatives
1	Mental disorders/problems (e.g., depression, anxiety, eating disorders)	Sexual and reproductive health, including adolescent pregnancy prevention	Mental health	Skin diseases	Adolescent fertility
2	Weight status (underweight, overweight, obesity)	HIV/AIDS	Sexual behaviors that contribute to HIV infection, other sexually transmitted infections, and unintended pregnancy	Road injury	Child marriage,^a^ education,^a^ violence,^a^ weight status^a^
3	Having support from parents	Sexually transmitted infections	Injury, including road traffic injury	Anxiety disorders	
4	Interpersonal violence (e.g., gang violence, bullying, war),^a^ Social media/Internet^a^	Tobacco	Cause-specific mortality,^a^ education (e.g., secondary education completion rate)^a^	Depressive disorders	
5		Substance use		Childhood behavioral disorders	
6	For adolescents to know about sexual health (birth control and family planning, abortion, prevention of diseases passed through sexual activity)	Mental health,^a^ nutrition^a^	Quality health service availability and access (including barriers)	Migraine	Contraception,^a^ tobacco use^a^
7	Skin diseases (e.g., dermatitis, acne)		Physical activity	Self-harm	
8	Sleep	Alcohol	Violence	Iron-deficiency anemia	Alcohol use,^a^ all-cause mortality,^a^ employment^a^
9	Using alcohol	Physical activity	Alcohol use	Drowning	
10	Using cigarettes or tobacco	Violence	Drug use,^a^ noncommunicable diseases^a^	Drug use	

a Same number of responses/inclusions, resulting in the same ranking.

RMNCAH Policy Survey results of 148 countries revealed that 135 had adolescents cited as a specific target group in a national policy or guideline for sexual and reproductive health, 134 for HIV/AIDS, and 130 for sexually transmitted infections (Table 2 and Appendix, p. 8).

Seventy professionals working in adolescent health in 21 countries responded to the rapid assessment tool. Most important health areas identified included mental health (n = 53), sexual behaviors (n = 50), injury (n = 39), cause-specific mortality, education (both n = 37), and quality health care services (n = 35) (Table 2 and Appendix, p. 13).

3. The adolescent burden of disease

Our analysis showed that across all 28 groups by sex, age (10–14 and 15–19 years), and modified WHO region, road injury, self-harm, and drowning were the most common causes contributing to adolescent mortality, included in 27, 15, and 12 groups, respectively. Most common causes of the nonfatal adolescent disease burden were skin diseases (included in 28 of 28 groups), anxiety disorders (24 groups), depressive disorders (21 groups), childhood behavioral disorders (20 groups), and migraine (17 groups). Iron deficiency was the most important risk factor contributing to adolescent DALYs and included in 8 of 28 sex/age/regional groups, whereas alcohol and drug use were the greatest contributors to adult DALYs, each included in 7 of 14 subgroups (Table 2 and Appendix, p. 15–16).

4. Adolescent health areas included in existing measurement initiatives

A total of 15 measurement initiatives and indicator compilations met our inclusion criteria (Table 3). Measurement areas represented in more than half of the 15 initiatives included adolescent fertility (12 initiatives), child marriage, education, violence, and weight status (all in eight initiatives; Table 2 and Appendix, p. 18–19).

### Core, expanded, and context-specific areas for adolescent health measurement

In total, we identified 99 health areas falling under the six domains in our adolescent health measurement framework, aligned with health areas of existing frameworks and across the four inputs. All areas are listed in Table 1, along with the frequency of inclusion in the four inputs, whereby "HI" represents a high frequency, "ME" represents a medium frequency, "LO" represents a low frequency, and an empty cell represents either a very low frequency or noninclusion of the measurement area in the specific input.

**Table 3:** Measurement initiatives and indicator compilations included^a^

Global indicator framework for the sustainable development goals and targets of the 2030 agenda for sustainable development^b^
The Lancet Commission on Adolescent Health and Wellbeing^c^
Indicator and Monitoring Framework for the Global Strategy for Women's, Children's, and Adolescents' Health (2016–2030)^d^
Countdown to 2030^e^
Family Planning 2020^f^
Adolescent Country Tracker^g^
Global Reference List of 100 core health indicators^h^
Global Reference List of Health Indicators for Adolescents (aged 10–19 years)^i^
Core Indicators for Adolescent Health: A Regional Guide (Eastern Mediterranean Regional Office)^j^
Commonwealth Youth Development Index^k^
INSPIRE Indicator Guidance and Results Framework^l^
Monitoring and Evaluation Guidance for School Health Programmes^m^
Measuring the Education Sector response to HIV and AIDS: Guidelines for the construction and use of core indicators^n^
UNECE Monitoring Framework for the ICPD Programme of Action beyond 2014^o^
WHO's 13th General Programme of Work Impact Framework^p^

a The Measurement of Mental Health among Adolescents at the Population Level (MMAP) initiative (https://data.unicef.org/topic/child-health/mental-health/mmap/) was also considered; however, at the time of our review, indicators were not finalized. Nine indicators are proposed now.

b United Nations. General Assembly. A/RES/71/313. Global indicator framework for the Sustainable Development Goals and targets of the 2030 Agenda for Sustainable Development. New York, USA: United Nations, 2017.

c Patton GC, Sawyer SM, Santelli JS, et al. Our future: A Lancet commission on adolescent health and wellbeing. *The Lancet* 2016; 387(10,036): 2423-78.

d Every Woman Every Child. Indicator and Monitoring Framework for the Global Strategy for Women's, Children's and Adolescents' Health 2016–2030. New York, 2016.

e Countdown to 2030. Tracking progress towards universal coverage for reproductive, maternal, newborn, and child health. *The Lancet* 2018; 391(10,129): 1538–48.

f Family Planning 2020. FP 2020. 2018. https://www.familyplanning2020.org/ (accessed December 4, 2019).

g UNICEF. Adolescent Country Tracker. 2018. https://data.unicef.org/resources/adolescent-country-tracker/ (accessed December 4, 2019).

h World Health Organization. Global Reference List of 100 Core Health Indicators (plus health-related SDGs). Geneva, Switzerland, 2018.

i World Health Organization. Global Reference List of Health Indicators for Adolescents (aged 10–19 years). Geneva, Switzerland, 2015.

j World Health Organization Regional Office for the Eastern Mediterranean. Core indicators for adolescent health: a regional guide. Cairo, Egypt: World Health Organization. Regional Office for the Eastern Mediterranean, 2014.

k The Commonwealth. The Commonwealth Youth Development Index. 2016. https://thecommonwealth.org/youthdevelopmentindex (accessed April 7, 2020).

l United Nations Children's Fund. INSPIRE Indicator Guidance and Results Framework - Ending Violence Against Children: How to define and measure change. New York, USA: UNICEF, 2018.

m UNESCO. Monitoring and Evaluation Guidance for School Health Programs. Paris, France: UNESCO, 2014.

n UNESCO. Measuring the education sector response to HIV and AIDS. Guidelines for the construction and use of core indicators. Paris, France: UNESCO, 2013.

o UNECE and UNFPA. UNECE Monitoring Framework for the ICPD Programme of Action beyond 2014. Geneva and Istanbul: UNECE and UNFPA, 2018.

p World Health Organization. WHO 13th General Programme of Work (GPW 13) Impact Framework: Targets and indicators. 2018. https://www.who.int/about/what-we-do/GPW13_WIF_Targets_and_Indicators_English.pdf (accessed April 7, 2020).

In the first survey round, 12 or more (>70%) of the 17 GAMA experts considered 33 health areas to be “core” measurement areas relevant for all adolescents globally (Table 1). Of those, the following five measurement areas were considered “core” by all (100%) GAMA experts: adolescent health policies and plans, contraception, HIV/AIDS, interpersonal violence, and sexual violence. In addition, more than 70% of GAMA experts considered two areas to be context-specific measurement areas: social and cultural norms and Vitamin A deficiency. Given the high level of consensus, GAMA experts agreed to limit the number of core areas to those with more than 70% agreement in the first survey round and to not perform a second round. However, areas which >50% but <70% of experts defined as “core” were further discussed over email, followed by a face-to-face meeting where GAMA experts agreed to include those as “expanded” measurement areas. It was also agreed that measurement areas which >50% of experts defined as “context-specific”, whereas the remaining experts chose “core” should be labeled as “context-specific”. This approach resulted in a total of 19 expanded and six context-specific measurement areas (Table 1).

## Discussion

The GAMA Advisory Group identified 33 core, 19 expanded, and 6 context-specific adolescent health measurement areas across six domains. To our knowledge, our approach was the first one aiming to concentrate measurement around comprehensively and systematically identified priority areas, considering inputs from the most important stakeholders. We strongly believe that this was a necessary first step toward focused, consistent global measurement of the most important adolescent health issues, better tracking of progress and guiding investments to improve adolescent health.

Our approach allowed us to identify where health areas of importance to adolescents, according to youth representatives and countries, and causing the greatest disease burden align with current measurement efforts. This was the case, for example, for several areas around substance use, weight status, sexual and reproductive health, and injury-related health areas. However, despite good coverage of actual measurement, the indicators and proposed measurement details sometimes vary greatly across initiatives. For instance, while adolescent fertility is included in 12 of the 15 initiatives reviewed with most initiatives proposing the indicator “adolescent birth rate,” indicator definitions are often different, or lack clarity in measurement description, calling for better alignment and measurement guidance [7].

Our approach also revealed measurement gaps. Mental disorders, for example, were considered a very important health area for adolescents by youth representatives, country professionals, and cause a large burden. However, mental disorders in a broad sense were only included in one [33], and depression as a specific health outcome in two of the 15 measurement initiatives reviewed [34,35]. To address this gap, the Measurement of Mental Health among Adolescents at the Population Level initiative, led by UNICEF, has defined core indicators in the area of adolescent mental health [36]. Work to validate them and to develop population-level data collection tools is underway, feeding into GAMA's efforts to fill gaps and improve the measurement of the most pertinent adolescent health issues in countries.

There was also consensus between country focal points and youth representatives that the quality of health care services was important. This measurement area is currently lacking consistent definitions and measurement not only in adolescent but also in maternal, newborn, and child health. A Think Tank Group, led by WHO, has recently published recommendations for standardizing “Effective Coverage” measurement among these population groups [37], which GAMA will also build on.

Additional health areas deemed important for adolescent health by youth representatives, yet not well captured in measurement initiatives, are sleep and support from parents. Although questions around both are included in international school surveys [38,39], consistent definitions are lacking, and further research is needed. This is also the case in the areas of skin diseases and migraine—both important according to our disease burden analysis, yet without any indicator across the 15 initiatives reviewed.

Overall, our review of measurement initiatives showed that current adolescent health measurement has a strong focus on health behaviors and risks and on health outcomes and conditions, whereas measurement of systems performance and interventions is weak. This may be driven by adolescent health measurement growing from existing structures, such as traditional population behavior surveillance systems, and by measuring what can be measured more easily, whereas gaps remain in equally important areas that are harder to measure [5]. Measurement of interventions is hampered by the currently sparse evidence related to their effectiveness, particularly in low- and middle-income countries [40]. There is an urgent need for greater investment in determining what works to improve adolescent health [41], so that countries can act to improve the health of their adolescents.

Collecting information through four critical inputs enabled the GAMA experts to make an informed decision on what the focus areas for global adolescent health measurement should be, leading to clear consensus in the selection of 33 core measurement areas. These are the areas of concentrated focus of GAMA's work in the next phase that has already begun, during which—in collaboration with other stakeholders—priority indicators are being selected, measurement guidance developed and globally promoted for use in countries [11,12]. Indicator selection for both expanded and context-specific areas will occur once this task is finalized for core areas and will be done together with countries. All selected measurement areas and indicators will be revisited regularly. For example, iron deficiency [9] and child marriage [42]—both currently labeled as context specific—might need to be elevated to be core measurement areas eventually, as they become of increased global concern, including through migration and globalization. Moreover, well-being was identified as a domain in our adolescent health measurement framework, but no measurement area under this domain was selected as core. This is being revisited and refined at GAMA's current stage of indicator selection, including in light of a new framework for adolescent well-being that identified five interconnected domains [43], published after the current priority areas had been selected, and building on existing frameworks such as the one on child development [44]. Collaborative work is underway to align and interlink these domains with GAMA's measurement framework domains and to develop a way forward for adolescent well-being measurement. This work will be described in a background article that will inform multistakeholder consultations to be held in April 2021 on policy and programmatic implications of the framework for adolescent well-being.

Our study has limitations. First, to collect input from young people, we did not survey adolescents aged 10–19 years directly. This decision was based on considering several potential issues, including ethical issues around obtaining consent [45]; potential difficulties of young adolescents to answer our online questions that require some health and general literacy; and difficulties in reaching some adolescent groups, including the most vulnerable, through an online survey. Instead, we chose to survey a sample of international youth representatives aged 18–29 years, assuming they would be well placed to represent the voices of many adolescent groups. This sample was unintentionally skewed toward female participants and by country and potentially toward those already working in the health or policy domains or with easy Internet access, which might have biased our results. However, provided the broad range of adolescent health issues and determinants in the answers provided by the responding youth representatives of over 60 countries across all regions, we believe that this input was valuable and important to our decision-making process. Going forward, GAMA's continued work will consider further engagement of young people and adolescents themselves where appropriate. Second, our input from countries was based on the RMNCAH Policy Survey that is designed for a different purpose, as well as on a convenience sample of professionals working in adolescent health in 21 countries responding to our rapid assessment tool. Responses are therefore not representative of all countries. However, provided the inclusion of countries of all income levels, we assume the variety of adolescent health issues across different settings is well captured. Third, for our analysis of the adolescent disease burden, we used highly modeled estimates [21,22] that are based on sometimes weak underlying country data or on assumptions where no country data exists [9,46]. However, these data are currently the best available estimates to reflect the disease burden of populations and are useful to identify health priorities. Fourth, when combining our four inputs and aligning the health areas across the inputs and with health areas of existing frameworks, it was sometimes inevitable to create categories of varying width or with some overlap. For example, although different types of injuries are included in the Global Health Estimates framework [21], such as road traffic or drowning, these were combined in one category in the survey for youth representatives and in the rapid assessment tool for countries. Therefore, when mapping health areas across inputs, we had to distribute survey responses across the different types of injury, which may not have done justice to each type of injury individually. However, during GAMA's current phase of indicator selection, for broad core measurement areas such as injuries, indicators around all individual injury types are being reconsidered. Fifth, our selection of priority areas may not reflect the needs of all adolescent populations in all settings because it is intentionally limited in number. However, priority setting approaches in health always reflect a compromise between health needs and limited resources in countries that GAMA is well aware of [13]. Sixth, although we used a systematic approach to priority setting, the final selection of priority health areas was done anonymously by only 17 experts. Although these GAMA experts represent all world regions and have been competitively selected to also represent a variety of health topics, they may not be representative of all experts in adolescent health measurement. Furthermore, although anonymity has the advantage of genuine and unbiased selection, we could not analyze the voting results by any variables such as sex, age, or country of the expert.

We took a systematic approach to concentrate measurement around priority areas of most potential utility for subsequent action. The selection of priority areas was based on an informed decision by international experts that considered and critically evaluated four inputs in a comprehensive way: perspectives of youth representatives, countries, the adolescent disease burden, and existing measurement efforts. Our analysis highlighted that current measurement does not necessarily reflect what should be measured according to youth representatives and country professionals and in terms of the health situation of adolescents. Important measurement gaps such as measurement of health service quality [37] for adolescents need to be filled, whereas in other areas, for example, adolescent sexual and reductive health [7], measurement needs to be better aligned to deliver useful data for action.

The selected priority areas for adolescent health measurement relate to the Sustainable Development Goals (SDGs) and their vision of a world free from poverty, hunger, and disease in two important ways. First, through promoting a concentrated focus on the core measurement areas, GAMA's work will support tracking progress and stimulate action toward many of the SDG subtargets, for example, target 3.4 to “reduce by one third premature mortality from noncommunicable diseases through prevention and treatment and promote mental health and well-being by 2030” in SDG 3 on good health and well-being but also in other areas such as SDG 1 (no poverty), SDG 4 (quality education), and SDG 5 (gender equality) [47]. Second, adolescents are recognized as playing a crucial role in achieving the SDGs, and therefore, it is essential that the SDGs are sensitive to the key issues they face [48]. Yet, the SDG framework has been criticized for its invisibility of adolescents and its lack of sufficient granularity of age and gender differences to enable measurement of progress among adolescents [49]. In using a systematic approach, GAMA's current effort highlights critical issues of relevance to adolescent health that are not in the SDGs, including, for instance, overweight and obesity, that urgently need to shift in the focus to be addressed appropriately. In that sense, GAMA's work is complementary to the SDGs in the area of adolescent health, calling for increased attention to and better measurement of these gaps, which will ultimately enable governments to better understand the specific issues of adolescents in their countries and implement specific interventions and policies to address those.

The next phase of GAMA's work, the selection of priority indicators within the identified priority areas has begun and will be completed in early 2021. Harmonized guidance to measure these indicators will be developed over the course of 2021, followed by supporting implementation in countries that will build on existing measurement systems. These tasks are being accomplished in collaboration with key partners to ensure that ultimately, all stakeholders collect the most relevant data on adolescents in a coherent way. We recognize that this will be a lengthy process spanning over several years; however, it will be critical to improve the health of adolescents and to achieve universal health coverage for all [50].
