# Supplementary material for: Priority Areas for Adolescent Health Measurement
Source: J Adolesc Health. 2021 May;68(5):888–98. doi: 10.1016/j.jadohealth.2020.12.127 (PMC8083105; doi:10.1016/j.jadohealth.2020.12.127)
Supplement: Appendix [file mmc1.docx]

**APPENDIX. Detailed information on the four inputs**

**1. Input from youth representatives through a survey with representatives of youth-led organizations**

The objective of the online survey was to collect perspectives of youth representatives on what they thought were the most important health issues and factors influencing the health of adolescents aged 10-19 years. The survey was administered to representatives of youth-led organizations aged 18-29 years working with adolescents aged 10-19 years.

The survey was distributed through the Partnership for Maternal, Newborn, and Child Health (PMNCH), the UN Major Group for Children and Youth, UNICEF regional adolescent focal points, representatives of other UN organizations, and other youth networks.

A total of 946 representatives of youth-led organizations participated. The survey was anonymous, and responses to participants’ demographics (sex and country) were voluntary. Of those that replied, 205 indicated they were male, while 556 indicated they were female. Participant’s countries included: Afghanistan, Algeria, Argentina, Australia, Azerbaijan, Bangladesh, Benin, Botswana, Cameroon, China, Czechia, Denmark, Egypt, Fiji, Gambia, Georgia, Germany, Ghana, Guatemala, Hungary, India, Indonesia, Iran (Islamic Republic of), Iraq, Israel, Jordan, Kenya, Kyrgyzstan, Lebanon, Lithuania, Madagascar, Malawi, Malaysia, Maldives, Mexico, Nepal, Netherlands, New Zealand, Nigeria, Pakistan, Panama, Philippines, Poland, Russian Federation, Rwanda, Senegal, Serbia, South Africa, Sri Lanka, State of Palestine, Syrian Arab Republic, Togo, Trinidad and Tobago, Tunisia, Uganda, Ukraine, United Kingdom, United Republic of Tanzania, United States of America, Uruguay, Yemen, Zimbabwe.

The questionnaire, the number of respondents, as well as the number of responses for each item (Table A1) are shown below.

**Survey questionnaire for representatives of youth-led organizations**

|  | **GAMA: Global Action for Measurement of Adolescent health**  **Priority areas for adolescent health measurement: Survey for youth representatives** |  |
| --- | --- | --- |

**Young people’s health**

**Introduction**

This survey is for young people aged 18-29 years who either work with adolescents aged 10-19 years, or represent their views in an organization. We kindly ask you to tell us **what is important for the health of adolescents aged 10-19 years**, based on your experience in working with adolescents or representing them.

Your answers will help the work of an initiative called the *Global Action for Measurement of Adolescent health* (GAMA). The GAMA works with WHO and other United Nations agencies to determine what is considered most important for the health of adolescents.

Once available, the survey results will be on the GAMA homepage: who.int/data/mncah/gama

**This survey consists of six questions and will take approximately 5 minutes to complete.** It is anonymous, and we will not ask for your name or any other confidential information. You can exit the survey at any time, but we would greatly appreciate if you could answer all the questions. By doing so, we assume that you give us your permission to use your responses for GAMA’s work.

Thank you very much for your participation!

**Questions**

*A. How old are you?*

*B. What do you think are the most important health issues for adolescents aged 10-19 years, based on your experience in working with them?* ***Tick up to 5*** *boxes from the list below.*

HIV/AIDS

Diseases, other than HIV/AIDS, that are passed through sexual activity (e.g. chlamydia, gonorrhoea, herpes, HPV, syphilis). These are called sexually transmitted infections.

Diseases, other than HIV/AIDS, that you **can** get from another person (e.g. flu, measles, pneumonia, tuberculosis, ebola)

Diseases that you **cannot** get from another person (e.g. diabetes, cancer, heart disease)

Diseases that you can get from insect bites or other animals (e.g. Malaria, Dengue, Zika, Rabies or Guinea worm disease)

Asthma

Weight status (underweight, overweight, obesity)

Anaemia (low iron)

Mental disorders/problems (e.g. depression, anxiety, eating disorders)

Suicide and self-harm

Interpersonal violence (e.g. gang violence, bullying, war)

Sexual violence

Injury (e.g. burns, falls, road traffic, drowning)

Skin diseases (e.g. dermatitis, acne)

Oral health

Problems with seeing or hearing

Migraine

Back or neck pain

Having a disability (e.g. not being able to walk)

Allergies

*C. What do you think are the most important factors that affect the health of adolescents aged 10-19 years, based on your experience in working with them? These factors may affect health in either positive or negative ways.* ***Tick up to 10*** *factors from the list below.*

Being in school (or having completed secondary education)

Having a job (or earning money)

Being from a poor family or neighborhood

Not knowing where your next meal will come from

Not having a regular place to sleep

Being a member of a community group

Being a member of a religious group

Living in a polluted environment (e.g. polluted air, pollution with waste)

Being part of a minority group (e.g. religious or ethnic minority; migrants; lesbian, gay, bisexual, transgendered or intersex; having a disability)

Having a boyfriend/girlfriend

Having support from parents

Having support from friends

Using alcohol

Using drugs

Using cigarettes or tobacco

Physical activity or exercise

Sitting too much

Sleep

Diet

Sexual health (birth control or family planning, abortion, prevention of diseases passed through sexual activity)

Getting married before age 18

Female genital mutilation or cutting

Social media / internet

Gaming

Advertisement (e.g. tobacco, food, body image)

Being able to use health facilities or services (location, transportation, hours of business)

Having free health care or services

0

Having good quality health care or services (safe, effective, confidential, young people are respected)

Having health services in schools

Being able to get vaccinations

Being able to get sexual health services (birth control or family planning, abortion, prevention of sexually transmitted infections, testing for HIV/AIDS)

For pregnant girls to be able to get care (during pregnancy and delivery, after childbirth)

Having access to toilets and clean water

For adolescents to know what is good and bad for their own health

For adolescents to know about sexual health (birth control and family planning, abortion, prevention of diseases passed through sexual activity)

*D. Are there any other health issues or factors that affect the health of adolescents aged 10-19 years that you think are important, based on your experience in working with them?*

*______________________________*

*E. What country do you live in?*

*F. What is your sex?*

Female Male Do not identify with either sex Do not want to answer

**Table A1.** Number of responses to items in the survey for representatives of youth-led organizations, by World Bank income group and total.

|  | ***Low income countries*** | ***Lower-middle income countries*** | ***Upper-middle income countries*** | ***High-income countries*** | ***Males*** | ***Females*** | ***TOTAL**** |
| --- | --- | --- | --- | --- | --- | --- | --- |
| ***Number of respondents*** | ***29*** | ***651*** | ***35*** | ***49*** | ***205*** | ***556*** | ***946*** |
| ***Most important health issues of adolescents*** | | | | | | | |
| HIV/AIDS | 13 | 78 | 6 | 7 | 39 | 65 | **133** |
| Diseases, other than HIV/AIDS, that are passed through sexual activity (e.g. chlamydia, gonorrhoea, herpes, HPV, syphilis) | 17 | 123 | 7 | 17 | 46 | 116 | **205** |
| Diseases, other than HIV/AIDS, that you can get from another person (e.g. flu, measles, pneumonia, tuberculosis, ebola) | 5 | 74 | 6 | 5 | 22 | 68 | **119** |
| Diseases that you cannot get from another person (e.g. diabetes, cancer, heart disease) | 2 | 63 | 4 | 5 | 22 | 54 | **101** |
| Diseases that you can get from insect bites or other animals (e.g. Malaria, Dengue, Zika, Rabies or Guinea worm disease) | 6 | 23 | 1 | 2 | 10 | 24 | **41** |
| Asthma | 1 | 24 | 1 | 4 | 4 | 25 | **40** |
| Weight status (underweight, overweight, obesity) | 4 | 328 | 16 | 25 | 78 | 293 | **448** |
| Anaemia (low iron) | 6 | 31 | 2 | 2 | 5 | 38 | **57** |
| Mental disorders/problems (e.g. depression, anxiety, eating disorders) | 10 | 404 | 23 | 37 | 124 | 346 | **604** |
| Suicide and self-harm | 2 | 185 | 12 | 29 | 69 | 155 | **279** |
| Interpersonal violence (e.g. gang violence, bullying, war) | 10 | 256 | 15 | 19 | 77 | 217 | **393** |
| Sexual violence | 21 | 158 | 12 | 14 | 62 | 141 | **262** |
| Injury (e.g. burns, falls, road traffic, drowning) | 3 | 114 | 5 | 3 | 32 | 94 | **154** |
| Skin diseases (e.g. dermatitis, acne) | 5 | 254 | 4 | 6 | 47 | 223 | **333** |
| Oral health | 2 | 60 | 6 | 4 | 23 | 50 | **96** |
| Problems with seeing or hearing | 3 | 152 | 9 | 5 | 35 | 133 | **212** |
| Migraine | 0 | 55 | 2 | 2 | 11 | 47 | **78** |
| Back or neck pain | 0 | 121 | 4 | 0 | 32 | 91 | **158** |
| Having a disability (e.g. not being able to walk) | 3 | 73 | 2 | 6 | 25 | 60 | **105** |
| Allergies | 1 | 128 | 4 | 3 | 41 | 96 | **162** |
| ***Most important factors affecting the health of adolescents*** | | | | | | | |
| Being in school (or having completed secondary education) | 11 | 93 | 10 | 12 | 41 | 85 | **142** |
| Having a job (or earning money) | 5 | 78 | 5 | 5 | 41 | 52 | **106** |
| Being from a poor family or neighborhood | 11 | 146 | 5 | 15 | 48 | 126 | **196** |
| Not knowing where your next meal will come from | 5 | 29 | 2 | 9 | 15 | 31 | **54** |
| Not having a regular place to sleep | 1 | 39 | 2 | 10 | 14 | 40 | **63** |
| Being a member of a community group | 4 | 79 | 3 | 3 | 33 | 57 | **102** |

|  | ***Low income countries*** | ***Lower-middle income countries*** | ***Upper-middle income countries*** | ***High-income countries*** | ***Males*** | ***Females*** | ***TOTAL**** |
| --- | --- | --- | --- | --- | --- | --- | --- |
| Being a member of a religious group | 3 | 31 | 1 | 2 | 13 | 25 | **42** |
| Living in a polluted environment (e.g. polluted air, pollution with waste) | 2 | 176 | 6 | 14 | 52 | 147 | **223** |
| Being part of a minority group (e.g. religious or ethnic minority; migrants; lesbian, gay, bisexual, transgendered or intersex; having a disability) | 4 | 75 | 11 | 15 | 35 | 68 | **117** |
| Having a boyfriend/girlfriend | 2 | 95 | 3 | 5 | 40 | 66 | **118** |
| Having support from parents | 7 | 310 | 13 | 21 | 67 | 282 | **413** |
| Having support from friends | 2 | 161 | 6 | 10 | 52 | 125 | **211** |
| Using alcohol | 2 | 271 | 9 | 11 | 80 | 209 | **328** |
| Using drugs | 5 | 181 | 6 | 13 | 71 | 131 | **234** |
| Using cigarettes or tobacco | 5 | 270 | 3 | 14 | 81 | 209 | **323** |
| Physical activity or exercise | 6 | 252 | 10 | 16 | 73 | 211 | **315** |
| Sitting too much | 1 | 194 | 7 | 5 | 60 | 145 | **233** |
| Sleep | 2 | 276 | 9 | 12 | 70 | 228 | **330** |
| Diet | 4 | 249 | 7 | 13 | 54 | 219 | **304** |
| Sexual health (birth control or family planning, abortion, prevention of diseases passed through sexual activity) | 10 | 207 | 14 | 16 | 61 | 185 | **280** |
| Getting married before age 18 | 11 | 64 | 3 | 3 | 29 | 48 | **92** |
| Female genital mutilation or cutting | 4 | 10 | 0 | 0 | 12 | 13 | **33** |
| Social media / internet | 14 | 292 | 16 | 21 | 89 | 252 | **393** |
| Gaming | 3 | 38 | 2 | 2 | 18 | 28 | **50** |
| Advertisement (e.g. tobacco, food, body image) | 6 | 98 | 6 | 7 | 31 | 90 | **139** |
| Being able to use health facilities or services (location, transportation, hours of business) | 6 | 84 | 9 | 10 | 33 | 73 | **126** |
| Having free health care or services | 7 | 147 | 5 | 10 | 36 | 132 | **187** |
| Having good quality health care or services (safe, effective, confidential, young people are respected) | 11 | 193 | 11 | 17 | 51 | 181 | **265** |
| Having health services in schools | 9 | 72 | 8 | 7 | 24 | 71 | **114** |
| Being able to get vaccinations | 3 | 78 | 5 | 6 | 26 | 67 | **110** |
| Being able to get sexual health services (birth control or family planning, abortion, prevention of sexually transmitted infections, testing for HIV/AIDS) | 10 | 131 | 11 | 17 | 48 | 124 | **194** |
| For pregnant girls to be able to get care (during pregnancy and delivery, after childbirth) | 12 | 69 | 4 | 6 | 23 | 66 | **104** |
| Having access to toilets and clean water | 4 | 93 | 3 | 9 | 29 | 83 | **127** |
| For adolescents to know what is good and bad for their own health | 11 | 243 | 16 | 11 | 72 | 209 | **317** |
| For adolescents to know about sexual health (birth control and family planning, abortion, prevention of diseases passed through sexual activity) | 10 | 283 | 12 | 19 | 74 | 250 | **352** |

*total includes respondents not indicating country and/or sex

**2.a Analysis of data collected through the Reproductive, Maternal, Newborn, Child and Adolescent Health (RMNCAH) Policy Survey**

The objective of the RMNCAH Policy survey was to track country progress in adopting WHO recommendations in national health policies, strategies and guidelines related to RMNCAH.^1^ With our analysis, we aimed to determine the frequency with which adolescents are cited as a specific target group for defined interventions/activities in a national policy/guideline for 14 specific, pre-defined health issues.

Survey administration was coordinated by WHO, through the WHO country offices and, where relevant, UNICEF and UNFPA country offices, in close collaboration with the Ministry of Health. Ministries of Health of the respective countries identified relevant focal points from different units to lead and/or assist with completion of the survey. The survey was administered using an online tool for which training materials were also provided.

At the time of our analysis, a total of 148 countries had completed the survey, including: Afghanistan, Albania, Algeria, Angola, Antigua and Barbuda, Argentina, Armenia, Australia, Austria, Azerbaijan, Bangladesh, Barbados, Belize, Benin, Bhutan, Bolivia (Plurinational State of), Botswana, Brazil, Brunei Darussalam, Bulgaria, Burkina Faso, Burundi, Cambodia, Cameroon, Chad, Chile, China, Colombia, Congo, Cook Islands, Costa Rica, Côte d’Ivoire, Croatia, Cuba, Cyprus, Czechia, Democratic Republic of the Congo, Denmark, Djibouti, Dominica, Dominican Republic, Ecuador, Egypt, El Salvador, Equatorial Guinea, Eritrea, Estonia, Eswatini, Finland, France, French Polynesia, Gambia, Georgia, Ghana, Grenada, Guam, Guatemala, Guinea, Guinea-Bissau, Guyana, Haiti, Honduras, India, Indonesia, Iraq, Israel, Italy, Jordan, Kazakhstan, Kenya, Kyrgyzstan, Lao People’s Democratic Republic, Latvia, Lebanon, Lesotho, Liberia, Lithuania, Luxembourg, Madagascar, Maldives, Mali, Malta, Marshall Islands, Mauritania, Mauritius, Mexico, Micronesia (Federated States of), Monaco, Mongolia, Morocco, Mozambique, Myanmar, Namibia, Nepal, Nicaragua, Niger, Nigeria, North Macedonia, Norway, Oman, Pakistan, Palau, Panama, Paraguay, Peru, Philippines, Poland, Portugal, Republic of Moldova, Romania, Russian Federation, Rwanda, Saint Kitts and Nevis, San Marino, Saudi Arabia, Senegal, Serbia, Sierra Leone, Singapore, Slovakia, Slovenia, Somalia, South Africa, South Sudan, Spain, Sri Lanka, State of Palestine, Sudan, Suriname, Sweden, Syrian Arab Republic, Tajikistan, Thailand, Togo, Trinidad and Tobago, Turkey, Turkmenistan, Uganda, United Republic of Tanzania, Uruguay, Uzbekistan, Vanuatu, Venezuela (Bolivarian Republic of), Viet Nam, Wallis and Futuna Islands, Yemen, Zambia, Zimbabwe.

The number of positive answers of countries responding to the question *“Are adolescents cited as a specific target group for defined interventions/activities in a national policy/guideline for the following health issues?”* are shown in Figure A1 below. The full RMNCAH Policy Survey questionnaire is available elsewhere.^2^

*including adolescent pregnancy prevention

**e.g. lower respiratory infections, meningitis, diarrhoea

**Figure A1.** Number of positive answers of a total of 148 countries responding to the question *“Are adolescents cited as a specific target group for defined interventions/activities in a national policy/guideline for the following health issues?”* of the Reproductive, Maternal, Newborn, Child and Adolescent Health (RMNCAH) Policy Survey. Answers were pre-defined.

**2.b Rapid assessment tool for countries**

The objective of the rapid assessment tool for countries was to collect perspectives of country adolescent health focal points and policy makers on what they thought were the most important areas for adolescent health measurement in their country.

The paper-based survey was distributed at two WHO regional adolescent health workshops (one in the WHO South-East Asia region, and one in the WHO African region), as well as through personal networks of the GAMA experts. Respondents were asked to select a maximum of ten of 34 pre-defined health areas that they thought were most important for routine adolescent health measurement in their country. In an open-ended question, they could also identify any other priority area for adolescent health measurement that should be considered.

Of a total of 104 questionnaires distributed, 70 (67.3%) were filled correctly and were used in our analysis. Incorrectly filled questionnaires included those where respondents selected more than ten of the health areas listed. The 70 respondents included adolescent health focal points from Ministries of Health, Ministries of Education, national NGOs, UN country offices, academic institutions, and national, independent consultants, from the following 21 countries: Bangladesh, Bhutan, Chile, China, Ethiopia, Kenya, Iran (Islamic Republic of), Malawi, Maldives, Myanmar, Nepal, Nigeria, India, South Africa, Sri Lanka, Thailand, Timor-Leste, Uganda, United Kingdom, United Republic of Tanzania, Zambia.

The assessment tool, as well as the number of responses to items in the tool (Table A2) are shown below.

**Rapid assessment tool for countries**

|  | **GAMA: Global Action for Measurement of Adolescent health**  **Priority areas for adolescent health measurement: Informal country questionnaire** |  |
| --- | --- | --- |

**Introduction**

In 2018, WHO, in collaboration with the UN H6+ partnership agencies (UNAIDS, UNESCO, UNFPA, UNICEF, UN Women, the World Bank, and the World Food Programme (WFP)), has established the *Global Action for Measurement of Adolescent health* (GAMA) Advisory Group.

The goals of GAMA’s work are:

- To provide technical guidance to WHO, UN H6+ agencies, and other relevant measurement groups to **define a core set of adolescent health indicators**, for the purpose of harmonizing efforts around adolescent health measurement and reporting;
- To promote **harmonised guidance** for adolescent health measurement, supporting countries and technical organizations in collecting useful data to track progress in the improvement of adolescent health.

As part of this work, GAMA is currently defining priority areas for adolescent health measurement. With this rapid questionnaire, we seek informal input from countries on **what should be priority areas for adolescent health measurement**.

Thank you very much for your participation!

**Questions**

*A. What country do you work in?*

*___________________________________________________________________*

*B. What Sectoral Ministry/Organization do you work for?*

Ministry of Health

Ministry of Education

Ministry of Youth

Other Ministry (other than Health, Education, Youth, e.g. Gender & Women)

NGO

WHO country office

UN agency country office (other than WHO)

Other (please specify below)

*______________________________________________*

*C. Please select* ***a maximum of 10*** *of the health areas listed below (with boxes next to them for marking) that you think are* ***most important for routine adolescent health measurement in your country****.*

**Mortality**

Overall (all-cause) mortality

Cause-specific mortality

**Morbidity**

HIV/AIDS

Communicable diseases other than HIV/AIDS

Non-communicable diseases

Nutritional status (weight; micro-nutrient deficiencies)

Anaemia

Mental health

**Other Health-related Outcomes/Conditions**

Adolescent fertility rate

Genital mutilation/cutting

**Health behaviours and health risks**

Alcohol use

Drug use

Tobacco use

Dietary behaviour

Physical activity

Violence

Injury, including road traffic injury

Sexual behaviours that contribute to HIV Infection, Other STI, and unintended

Pregnancy

Self-harm

**Health determinants**

Population (e.g. % of adolescents/youth of overall population)

Education (e.g. secondary education completion rate)

Poverty

Employment among adolescents and youth

Child marriage

Protective factors (e.g. parent or peer support)

Gender norms (including sex ratio in adolescents)

Adolescent-specific considerations for programming in humanitarian and

fragile settings

**Health System Outputs**

Quality health service availability and access (including barriers)

Health service utilization

Financial protection for health (health insurance and no fee for services)

**Intervention Areas**

Screening (e.g. vision and hearing)

Vaccination (e.g. HPV; tetanus toxoid)

Health services (e.g. for communicable diseases; nutrition; sexual and reproductive health, including menstrual hygiene; contraception; safe abortion and maternity care; mental health; trauma)

Information & skills (e.g. comprehensive sexuality education; prevention of mosquito-borne disease; promotion of physical education; road safety)

*C.1. Are there any other priority areas for adolescent health measurement that should be considered?*

________________________________________________________________________

*D. Does your country have an established system to monitor these health areas routinely?*

Yes, for ALL areas selected

Only for some of the areas selected (Please specify which ones)

________________________________________________________________________

No, for NONE of the areas selected

Don’t know

**Table A2.** Number of responses to items in the rapid country assessment tool.

| ***Number of respondents*** | ***70*** |
| --- | --- |
| ***Mortality*** | |
| Overall (all-cause) mortality | 10 |
| Cause specific mortality | 37 |
| ***Morbidity*** | |
| HIV/AIDS | 14 |
| Communicable diseases other than HIV/AIDS | 4 |
| Noncommunicable diseases | 26 |
| Nutritional status (weight; micro-nutrient deficiencies) | 10 |
| Anemia | 20 |
| Mental health | 53 |
| ***Other health-related outcomes/conditions*** | |
| Adolescent fertility rate | 13 |
| Genital mutilation/cutting | 2 |
| ***Health behaviours and risks*** | |
| Alcohol use | 28 |
| Drug use | 26 |
| Tobacco use | 22 |
| Dietary behaviour | 17 |
| Physical activity | 30 |
| Violence | 29 |
| Injury, including road traffic injury | 39 |
| Sexual behaviours that contribute to HIV infection, other STI, and unintended pregnancy | 50 |
| Self-harm | 4 |
| ***Health determinants*** | |
| Population (e.g. % of adolescents/youth of overall population) | 14 |
| Education (e.g. secondary education completion rate) | 37 |
| Poverty | 12 |
| Employment among adolescents and youth | 12 |
| Child marriage | 16 |
| Protective factors (e.g. parent or peer support) | 25 |
| Gender norms (including sex ratio in adolescents) | 3 |
| Adolescent-specific considerations for programming in humanitarian and fragile settings | 1 |
| ***Health system outputs*** | |
| Quality health service availability and access (including barriers) | 35 |
| Health service utilization | 20 |
| Financial protection for health (health insurance and no fee for services) | 9 |
| ***Intervention areas*** | |
| Screening (e.g. vision and hearing) | 9 |
| Vaccinaton (e.g. HPV; tetanus toxiod) | 4 |
| Health services (e.g. for communicable diseases; nutrition; sexual and reproductive health, including menstrual hygiene; contraception; safe abortion and maternity care; mental health; trauma) | 23 |
| Information & skills (e.g. comprehensive sexuality education; prevention of mosquito-borne disease; promotion of physical education; road safety) | 6 |

**3. The adolescent burden of disease**

The objective of the analysis of the adolescent burden of disease was to identify the main causes of (1) adolescent mortality and (2) adolescent morbidity, as measured in Years Lost due to Disability (YLD). This analysis also sought to identify (3) the main risk factors for Disability-Adjusted Life Years (DALYs) among adolescents, as well as (4) among adults, to account for future health loss through risk factors established during adolescence.

We used the WHO Global Health Estimates for the year 2016^3^ for the adolescent mortality and morbidity analysis, and the Global Burden of Disease estimates 2017^4^ for the risk factor analysis. To account for variations in burden across different groups, these analyses were disaggregated by sex, age group (for adolescents, 10-14 and 15-19 years, and for adults, under 50 years), as well as by modified WHO Region0F^[[1]](#footnote-1)^, whereby high-income countries were extracted from their region and combined in a separate group as in previous reports.^5^ This resulted in 28 sex/age/regional groups for adolescents, and 14 for adults.

For each sex/age/regional group, causes that contributed by more than 5% to the mortality or non-fatal disease burden of adolescents were listed. Risk factors that contributed by more than 5% to total adolescent or adult DALYs, and that are prevalent and modifiable through interventions during adolescence, were also included.

We conducted sensitivity analyses using – instead of the 5% threshold – a 10% and a 3% threshold. While using a 10% threshold would have led to inclusion of too few health areas (for example, only five causes contributed with over 10% to the morbidity burden across sex/age/regional groups), a 3% threshold would have identified nearly double the number of causes as compared to a 5% threshold, with several rather rare causes contributing only to one sex/age/regional group.

The results or our analysis are shown in Table A3 below.

**Table A3.** Number of inclusions of causes/risk factors contributing to over 5% of the (1) adolescent mortality burden, (2) the adolescent disease burden (as measured in Years Lost due to Disability – YLD), the (3) adolescent Disability-Adjusted Live Years (DALY) burden, and the adult DALY burden in sex/age/regional groups*.

| ***Cause contributed to over 5% of the mortality burden*** | ***Number of inclusions in sex/age/regional groups* (total=28 groups)*** |
| --- | --- |
| Road injury | 27 |
| Self-harm | 15 |
| Drowning | 12 |
| Interpersonal violence | 9 |
| Leukaemia | 9 |
| Lower respiratory infections | 8 |
| Congenital anomalies | 6 |
| Diarrhoeal diseases | 6 |
| Collective violence and legal intervention | 4 |
| HIV/AIDS | 4 |
| Maternal conditions | 4 |
| Meningitis | 4 |
| Tuberculosis | 3 |
| Brain and nervous system cancers | 2 |
| Malaria | 2 |
| Sickle cell disorders and trait | 2 |
| Cirrhosis of the liver | 1 |
| ***Cause contributed to over 5% of the non-fatal disease burden (as measured in YLD)*** | ***Number of inclusions in sex/age/regional groups* (total=28 groups)*** |
| Skin diseases | 28 |
| Anxiety disorders | 24 |
| Depressive disorders | 21 |
| Childhood behavioural disorders | 20 |
| Migraine | 17 |
| Iron-deficiency anaemia | 12 |
| Asthma | 7 |
| Autism and Asperger syndrome | 3 |
| Congenital anomalies | 2 |
| Uncorrected refractive errors | 2 |
| Drug use disorders | 1 |
| Preterm birth complications | 1 |
| ***Risk factor contributed to over 5% of the adolescent disease burden (as measured in DALY)*** | ***Number of inclusions in sex/age/regional groups* (total=28 groups)*** |
| Iron deficiency | 8 |
| Drug use | 3 |
| Alcohol use | 2 |
| Unsafe sex | 2 |
| Vitamin A deficiency | 1 |

| ***Risk factor contributed to over 5% of the adult disease burden (as measured in DALY)*** | ***Number of inclusions in sex/age/regional groups** (total=14 groups)*** |
| --- | --- |
| Alcohol use | 7 |
| Drug use | 7 |
| High LDL cholesterol | 1 |
| High body-mass index | 8 |
| High fasting plasma glucose | 2 |
| High systolic blood pressure | 4 |
| Iron deficiency | 1 |
| Smoking | 5 |
| Unsafe sex | 2 |

*the groups have been defined as follows: by sex (male; female), age (10-14; 15-19 years), and region (low- and middle income countries of the six WHO regions: (1) African Region; (2) Region of the Americas; (3) Eastern Mediterranean Region; (4) European Region; (5) South-East Asia Region; (6) Western Pacific Region; and (7) high-income countries).

**the groups have been defined as follows for the adults under 50 years: by sex (male; female), and region (low- and middle income countries of the six WHO regions: (1) African Region; (2) Region of the Americas; (3) Eastern Mediterranean Region; (4) European Region; (5) South-East Asia Region; (6) Western Pacific Region; and (7) high-income countries).

**4. Existing adolescent health measurement initiatives**

The objective of the review of existing adolescent health measurement initiatives and indicator compilations was to identify which measurement areas are currently covered by existing measurement efforts.

In order to be included, an initiative or an indicator compilation needed to (1) include recommendations about adolescent health measurement; (2) propose at least one indicator specifically including “adolescent”, “youth” or “young people”, or including the entire or part of the adolescent age range 10-19 years; and (3) be global or regional.

Adolescent health measurement initiatives and indicator compilations were identified through a review of existing reports,^6,7^ as well as through consultation of experts. Experts consulted included members of the GAMA Advisory Group, UN representatives, focal points from nine topic-specific WHO departments relevant for adolescent health, as well as WHO regional office focal points.

In total, we identified 15 current measurement initiatives and indicator compilations including adolescent health indicators, including the Global indicator framework for the Sustainable Development Goals and targets of the 2030 Agenda for Sustainable Development,^8^ The Lancet Commission on Adolescent health and wellbeing,^9^ the Indicator and Monitoring Framework for the Global Strategy for Women’s, Children’s, and Adolescents’ Health (2016-2030),^10^ Countdown to 2030,^11^ Family Planning 2020,^12^ the Adolescent Country Tracker,^13^ the Global Reference List of 100 core health indicators,^14^ the Global Reference List of Health Indicators for Adolescents (aged 10-19 years),^15^ the Core Indicators for Adolescent Health: A Regional Guide (EMRO),^16^ the Commonwealth Youth Development Index,^17^ the INSPIRE Indicator Guidance and Results Framework,^18^ the Monitoring and Evaluation Guidance for School Health Programmes,^19^ Measuring the Education Sector response to HIV and AIDS: guidelines for the construction and use of core indicators,^20^ the UNECE Monitoring Framework for the ICPD Programme of Action beyond 2014,^21^ and WHO’s 13^th^ General Programme of Work Impact Framework.^22^

The Measurement of Mental Health among Adolescents at the Population Level (MMAP) initiative^23^ was also considered and while nine indicators are proposed now, these were not finalised at the time of our review.

All initiatives and indicator compilations identified were reviewed as to which specific adolescent health measurement areas they covered. The results or this review are shown in Table A4 below.

**Table A4.** Number of inclusions of health areas in existing measurement initiatives and indicator compilations including adolescent health indicators.

|  | ***Number of initiatives this health area is included*** |
| --- | --- |
| ***Policies and programming*** | |
| Adolescent health policies/plans (availability, implementation, funding, M&E) | 4 |
| Adolescent health protective laws (availability, implementation, funding, M&E) | 2 |
| ***Systems performance and interventions*** | |
| Health service availability and access | 1 |
| Pregnancy and antenatal care | 2 |
| Health service quality | 1 |
| Health service use | 2 |
| Immunization | 2 |
| School health | 3 |
| Health education | 4 |
| Training/education in adolescent health for professionals | 2 |
| Social protection | 1 |
| Financial independency | 1 |
| Monitoring and surveillance | 3 |
| ***Health determinants*** | |
| Population | 3 |
| Education | 8 |
| Employment | 6 |
| Poverty | 3 |
| Being part of a vulnerable group (orphaned, out-of-school, migrant, minority etc) | 2 |
| Disaster risk reduction | 1 |
| Water, Sanitation and Hygiene | 1 |
| Child marriage | 8 |
| Child labour | 1 |
| Gender | 1 |
| Social support | 2 |
| ***Health behaviours and risks*** | |
| Weight status | 8 |
| Alcohol use | 6 |
| Substance use (general) | 3 |
| Substance use (other than alcohol and tobacco) | 2 |
| Tobacco use | 7 |
| Social media/internet | 2 |
| Dietary behaviour | 2 |
| Physical activity | 5 |
| Sedentary behaviour | 2 |
| Bullying | 3 |
| Sexual health | 4 |
| Reproductive health | 5 |
| Contraception | 7 |
| Menstruation | 1 |

|  | ***Number of initiatives this health area is included*** |
| --- | --- |
| ***Well-being*** | |
| Autonomy | 2 |
| Social connectedness | 1 |
| Wellbeing | 1 |
| ***Health outcomes and conditions*** | |
| ***Mortality*** | |
| All-cause mortality | 6 |
| ***Communicable, maternal, perinatal and nutritional conditions*** | |
| HIV/AIDS | 5 |
| Malaria | 1 |
| Worms | 1 |
| Maternal conditions | 2 |
| Iron-deficiency anemia | 3 |
| ***NCDs*** | |
| NCDs | 1 |
| Suicide | 3 |
| Mental disorders | 1 |
| Depression | 2 |
| Eye health | 1 |
| Hearing | 1 |
| Oral health | 1 |
| ***Injuries (Unintentional and intentional)*** | |
| Injury and violence combined | 1 |
| Road injury | 1 |
| Injury | 2 |
| Violence | 8 |
| Interpersonal violence | 3 |
| Sexual violence | 4 |
| ***Other health-related outcomes and conditions*** | |
| Female genital mutilation/cutting | 2 |
| Adolescent fertility | 12 |

**References**

1. World Health Organization. Maternal, newborn, child and adolescent health policy indicators. 2019. <https://www.who.int/maternal_child_adolescent/epidemiology/policy-indicators/en/> (accessed 4 October 2019).

2. World Health Organization. RMNCAH Policy Survey questionnaire. 2019. <https://www.who.int/docs/default-source/mca-documents/policy-survey-questionnaires/en-rmncah-policy-survey-final.pdf?sfvrsn=d91d9ec7_2> (accessed 1 July 2020).

3. World Health Organization. Global Health Estimates (GHE). <https://www.who.int/healthinfo/global_burden_disease/en/> (accessed 17 February 2020).

4. Institute for Health Metrics and Evaluation. Global Burden of Disease. <http://www.healthdata.org/gbd> (accessed 17 September 2019).

5. World Health Organization. Global Accelerated Action for the Health of Adolescents (AA-HA!): guidance to support country implementation. Geneva, Switzerland, 2017.

6. Azzopardi P, Kennedy E, Patton G. Data and indicators to measure adolescent health, social development and well-being. Florence, Italy, 2017.

7. Azzopardi PS, Hearps SJC, Francis KL, et al. Progress in adolescent health and wellbeing: tracking 12 headline indicators for 195 countries and territories, 1990-2016. *Lancet (London, England)* 2019; **393**(10176): 1101-18.

8. United Nations. General Assembly. A/RES/71/313. Global indicator framework for the Sustainable Development Goals and targets of the 2030 Agenda for Sustainable Development. New York, USA: United Nations, 2017.

9. Patton GC, Sawyer SM, Santelli JS, et al. Our future: a Lancet commission on adolescent health and wellbeing. *Lancet (London, England)* 2016; **387**(10036): 2423-78.

10. Every Woman Every Child. Indicator and Monitoring Framework for the Global Strategy for Women’s, Children’s and Adolescents’ Health 2016-2030. New York, 2016.

11. Countdown to 2030: tracking progress towards universal coverage for reproductive, maternal, newborn, and child health. *Lancet (London, England)* 2018; **391**(10129): 1538-48.

12. Family Planning 2020. FP 2020. 2018. <https://www.familyplanning2020.org/> (accessed 4 December 2019).

13. UNICEF. Adolescent Country Tracker. 2018. <https://data.unicef.org/resources/adolescent-country-tracker/> (accessed 4 December 2018).

14. World Health Organization. Global Reference List of 100 Core Health Indicators (plus health-related SDGs). Geneva, Switzerland, 2018.

15. World Health Organization. Global Reference List of Health Indicators for Adolescents (aged 10-19 years). Geneva, Switzerland, 2015.

16. World Health Organization Regional Office for the Eastern Mediterranean. Core indicators for adolescent health: a regional guide. Cairo, Egypt: World Health Organization. Regional Office for the Eastern Mediterranean, 2014.

17. The Commonwealth. The Commonwealth Youth Development Index. 2016. <https://thecommonwealth.org/youthdevelopmentindex> (accessed 7 April 2020).

18. United Nations Children's Fund. INSPIRE Indicator Guidance and Results Framework - Ending Violence Against Children: How to define and measure change. New York, USA: UNICEF, 2018.

19. UNESCO. Monitoring and Evaluation Guidane for School Health Programs. Paris, France: UNESCO, 2014.

20. UNESCO. Measuring the education sector response to HIV and AIDS. Guidelines for the construction and use of core indicators. Paris, France: UNESCO, 2013.

21. UNECE and UNFPA. UNECE Monitoring Framework for the ICPD Programme of Action beyond 2014. Geneva and Istanbul: UNECE and UNFPA, 2018.

22. World Health Organization. WHO 13th General Programme of Work (GPW 13) Impact Framework: Targets and indicators. 2018. <https://www.who.int/about/what-we-do/GPW13_WIF_Targets_and_Indicators_English.pdf> (accessed 7 April 2020).

23. UNICEF. Measurement of Mental Health Among Adolescents at the Population Level (MMAP). 2020. <https://data.unicef.org/topic/child-health/mental-health/mmap/> (accessed 2 July 2020).

1. Low- and middle income countries (LMICs) of the WHO African Region; LMICs of the WHO Region for the Americas; LMICs of the WHO Eastern Mediterranean Region; LMICs of the WHO European Region; LMICs of the WHO South-East Asia Region; LMICs of the WHO Western Pacific Region; and High-income countries. [↑](#footnote-ref-1)
